# Effects of Genistein on Lipid Metabolism, Antioxidant Activity, and Immunity of Common Carp (*Cyprinus carpio* L.) Fed with High-Carbohydrate and High-Fat Diets

**DOI:** 10.1155/2023/9555855

**Published:** 2023-03-31

**Authors:** Liping Yang, Mengjuan Zhao, Mingyu Liu, Wenlei Zhang, Shaoyang Zhi, Leya Qu, Jinrui Xiong, Luming Wang, Chaobin Qin, Guoxing Nie

**Affiliations:** College of Fisheries, Henan Normal University, No. 46 Jianshe Road, Xinxiang 453007, China

## Abstract

A 56-day feeding trial was conducted to investigate the effects of genistein on growth, lipid metabolism, antioxidant capacity, and immunity of common carp fed with high-carbohydrate or high-fat diets. Five diets were used to feed fish: control diet (5% fat; CO), high-fat diet (11% fat; HF), high-carbohydrate diet (45% carbohydrate; HC), and HF or HC diet with 500 mg/kg genistein (FG or CG). Results showed that final body weight (FW) and specific growth rate (SGR) were significantly reduced, but the supplementation with genistein resulted in higher values of FW and SGR than the HF or HC group. Both high carbohydrate and high fat belong to high-energy diets, which may promote lipid deposition. Genistein obviously decreased liver triglyceride (TG) content and alleviated hepatic fat vacuolation in the HF and HC groups. The expression of lipid metabolism genes (*cpt-1* and *atgl*) was markedly higher in the FG group than in the HF group. The lipid synthesis-related genes (*fas*, *acc*, and *pparγ*) were elevated in high-energy diets but recovered to the control level or reduced after genistein treatments. With respect to fatty acid transporter genes, *fatp* increased in the FG group, and *cd36* increased in the CG group. Furthermore, the antioxidant and immune indexes, such as total antioxidant capacity (T-AOC), glutathione peroxidase (GSH-PX), superoxide dismutase (SOD), acid phosphatase (ACP), and lysozyme (LZM) activities, were decreased, while malonate aldehyde (MDA) content, activities of alanine aminotransferase (ALT), and aspartate aminotransferase (AST) were enhanced in the HF and HC groups. The antioxidant and immunity values could be ameliorated by treatment with genistein. Moreover, the transcript levels of antioxidant-related genes (*cat*, *gr*, and *nrf2*) in the liver and anti-inflammatory factors (*tgf-β* and *il-10*) and *lyz* in the head kidney tissue were promoted, although the expression levels of proinflammatory factors (*tnf-α* and *il-6*) declined in the genistein supplementation group, which confirmed the antioxidant and immune-enhancing effects of genistein. Therefore, 500 mg/kg genistein could ameliorate the negative effects of high-energy diets on immunity.

## 1. Introduction

Carbohydrates, lipids, and protein are important energy sources in animals. Among them, carbohydrates and lipids are nonprotein energy sources [1]. In the intensive breeding mode, dietary high-energy sources have been widely used for protein sparing due to their higher availability and relatively lower price than fish meals [2]. However, intake of a high-energy diet can induce the symptoms of metabolic syndrome, such as nonalcoholic fatty liver disease (NAFLD) in mammals [3]. Moreover, obesity or dyslipidemia induced by high-energy diets has been found in fish, including Nile tilapia (*Oreochromis niloticus*) [4], pufferfish (*Takifugu fasciatus*) [5], medaka (*Oryzias latipes*) [6], blunt snout bream (*Megalobrama amblycephala*) [7], and grass carp (*Ctenopharyngodon idella*) [8]. It has been shown that diets with high-energy feed resulted in metabolic disturbances in fish. Long-term diets with high carbohydrates resulted in a significant deposition of fat in serum and the liver, as the carbohydrates can be converted into lipids through lipogenesis. Generally, herbivorous and omnivorous fish have a greater ability to convert carbohydrates to lipids, which has been studied in gibel carp (*Carassius gibelio*) [9], Nile tilapia [10, 11], and blunt snout bream [12]. In addition, lipid accumulation could be observed after chronic feeding with a high-fat diet of fish because fatty acid oxidation, as well as fatty acid transport capacity, is inhibited [13–15]. Furthermore, high-carbohydrate and high-fat diets can lead to oxidative stress [16–19] and poor immune function in fish [20–23]. Hepatic lipid dysregulation, oxidative stress, and proinflammatory cytokines act synergistically to trigger hepatic lipid accumulation [24, 25]. Our laboratory previously reported that lipid accumulation in Nile tilapia was caused by high carbohydrate or lipid levels in diets [1]. Therefore, it is imperative to find an effective treatment with a nutritional strategy to mitigate the negative effects of high-energy diets on fish.

Genistein (4,5,7-trihydroxyisoflavone) is one of the main isoflavones distributed in the germ of soybean, fava bean, and alfalfa, whose physiological actions have been widely confirmed in humans and animals. In addition to improving immunity in mammals fed with high-carbohydrate and high-fat diets, genistein has been shown to exert effects on oxidative stress and lipid and glucose metabolism [26–30]. Additionally, studies have indicated that the control of obesity by diets rich in soy isoflavones is mainly achieved by reducing blood triglyceride (TG) and total cholesterol (T-CHO) in broiler breeder hens and rabbits [31, 32]. Despite such beneficial effects, few studies have dealt with the function of dietary genistein with a high-energy diet in fish. In one of the studies, TG in serum of the genistein-injected group was remarkably downregulated in rainbow trout (*Oncorhynchus mykiss*) [33]. Our laboratory previously found that when low and medium doses of genistein (1 *μ*M and 10 *μ*M) are incubated with carp primary hepatocytes in vitro, the expression of lipid breakdown-related genes was enhanced [34]. In addition, adding an appropriate level of genistein (100, 500 mg/kg) to the basal feed was found to reduce the lipid synthesis, increase lipolysis, and improve both fish immunity and antioxidant capacity in common carp [35]. Preliminary studies have certified that appropriate concentrations of genistein pose beneficial effects on fat reduction, antioxidant capacity, and immunity. However, to date, the literature reports on the ability of genistein to mitigate the adverse impacts of high-carbohydrate and high-fat loads in aquatic animals are very scarce.

Common carp (*Cyprinus carpio* L.) is one of the most essential freshwater fish, widely cultured in China, with its aquaculture production reaching 2.9 million tons in 2020 (China Fishery Statistical Yearbook, 2021). However, with the rapid development of the aquaculture industry, there is an extensive use of high-energy diets leading to fat accumulation in carp. Therefore, it is urgent to find an additive that can improve the adverse impacts of high-carbohydrate and high-fat dietary load. In this study, five diets, the normal diet (CO, composed of 30% carbohydrates and 5% fat), the high-fat diet (HF, composed of 30% carbohydrates and 11% fat), the high-carbohydrate diet (HC, composed of 45% carbohydrates and 5% fat), the HF diet with 500 mg/kg genistein (FG), and the HC diet with 500 mg/kg genistein (CG), have been used to feed juvenile common carp. The eight-week breeding experiment was conducted to identify the effects of genistein on growth performance, lipid metabolism, antioxidant capacity, and immune response in common carp. The results will provide a certain theoretical and practical basis for the application of genistein in common carp.

## 2. Materials and Methods

### 2.1. Ethical Statement

All fish-related trials were executed in agreement with Laboratory Animals of Henan Normal University's protocols and procedures.

### 2.2. Feed Diets

The formulations and components of feed diets are listed in Table 1. Genistein with a purity of at least 98% was supplied by Dalian Meilun Biological Technology (Dalian, China). Five isonitrogenous experimental diets were designed, including a control diet (CO, composed of 30% carbohydrates, 5% fat, and 32% protein), high-fat diet (HF, composed of 30% carbohydrates, 11% fat, and 32% protein), high-carbohydrate diet (HC, composed of 45% carbohydrates, 5% fat, and 32% protein), HF with 500 mg/kg genistein (FG), and HC with 500 mg/kg genistein diet (CG) (Table 1). All nutrition ingredients of the diets were passed through a 60 *μ*m mesh before being thoroughly mixed with distilled water. Wet mixtures were extruded through the 1.5 mm diameters with a pelleting machine. After being air-dried at normal room temperature, the feed diets were kept at 4°C until used.

### 2.3. Fish Rearing and Experiment Design

Experimental fish were provided by Yanjin Aquaculture Company in Xinxiang, Henan Province, China. Before formal growth experimentation, all fish were temporarily acclimated for two weeks in the circulating aerated water in 180 L tanks at the College of Fishery, Henan Normal University. During an acclimation phase, all fish were hand-fed thrice daily by using the mix of the five experimental diets. After the acclimation, the fish fasted for 24 h, and the common carp (initial body weight was 6.36 ± 0.18 g) were selected and randomly divided into 15 tanks with 20 fish per tank in a recirculation aquaculture system. Each diet was assigned to 3 tanks, and the 15 tanks were distributed into CO, HF, HC, FG, and CG groups. The fish were fed with diets three times (08:30 h, 12:30 h, and 18:00 h) daily during the 8-week growth trial. During the experiment period, the principal parameters are as follows: the water temperature was maintained at about 25–28°C, dissolved oxygen range was 6.0 to 7.0 mg L^−1^, and pH range was 7.2 to 7.5 and under a natural photoperiod.

### 2.4. Sample Collection

After eight weeks of breeding experiments, the fish fasted for 24 h before collecting. Fish were under anesthetization with 55 mg/L MS-222 and individually weighed. Blood was immediately sampled from tail vessels with 6 fish per tank using 1.0 mL of heparinized syringes (Klmediacal, China), and the serum was obtained after centrifugation of treated samples (7000 × *g*, 10 min, and 4°C). After the dissection of fish, the hepatosomatic index and visceral somatic index were determined. Samples of the head kidney and liver tissues were obtained instantly after blood collection and then frozen with liquid nitrogen before being stored at −80°C prior to RNA isolation and enzyme activity detection. Fresh liver pellets were quickly collected from two fish per tank, and fixation in 4% paraformaldehyde was used for further histological observation.

### 2.5. Calculation of Growth Performance Indicators of Common Carp

Standard calculation formula was used to evaluate growth, feed utilization, visceral parameters, and survival rate, which were calculated according to the previous report [36, 37]. These indicators include the initial body weight (IW), final body weight (FW), weight gain rate (WGR, %), specific growth rate (SGR, % day^−1^), hepatosomatic index (HSI, %), visceral somatic index (VSI, %), and survival rate (SR, %).

### 2.6. Proximate Composition Analysis

According to the Weende feed analysis system, the proximate composition of experimental diet, fish, and muscle experiments was for chemical analysis. The moisture was determined by drying the samples to constant weight at 105°C [38]. The ash was estimated based on national standard specifications [38] and burned in a muffle furnace at a temperature of 550°C before being fully determined. The content of crude protein was determined using the Kjeldahl method, and crude lipid was measured with the Soxhlet extractor method, separately.

### 2.7. Serum Biochemical Parameter Analysis

Serum biochemicals include glucose (GLU), T-CHO, TG, low-density lipoprotein cholesterol (LDL-C), high-density lipoprotein cholesterol (HDL-C), aspartate transaminase (AST), alanine transaminase (ALT), alkaline phosphatase (AKP), acid phosphatase (ACP), and lysozyme (LZM) together with total antioxidant capacity (T-AOC), superoxide dismutase (SOD), glutathione peroxidase (GSH-PX), and malondialdehyde (MDA) that were detected by the experiment-related kits obtained from Nanjing Jiancheng Bioengineering Institution (Nanjing, China). All measurement steps were performed according to the instructions of relevant kit protocols. And the data were acquired with the automatic biochemical analyzer (AU-5800, Beckman).

### 2.8. Hepatic Lipid Metabolism, Immunity, and Antioxidant Enzyme Activities

To determine the lipid metabolism enzyme activity, liver tissue was manipulated according to the instructions of the kits. The content of T-CHO, TG, LDL-C, and HDL-C in the liver was analyzed following the method described by means of commercially produced kits (Jiancheng Biotech. Co., Nanjing, China). The enzyme activities of lipoprotein lipase (LPL) and fatty acid synthase (FAS) were measured by kits from Solarbio Science (Beijing, China).

To determine the immunity and antioxidant enzyme activities, liver tissue under ice bath was ground in 0.86% (*w*/*v*) physiological saline with a homogenizer and then centrifuged (2500 rpm with 10 minutes constant at 4°C). After that, AST, ALT, SOD, and MDA were detected using commercial assay kits (Jiancheng Biotech. Co., Nanjing, China).

The content of hepatic glycogen was measured following the manufacturer in the test kit instruction (Beijing Solarbio Science and Technology Co. Ltd., Beijing, China). In addition, a BCA protein quantitative detection kit was used to measure protein concentration.

### 2.9. Histological Observation

After the fixation of the liver tissue utilizing 4% paraformaldehyde up to 24 h, the samples were dehydrated through a progressive ethanol series and transparent by xylene and then embedded in paraffin wax. Hematoxylin and eosin (H&E) staining was conducted with 5 *μ*m consecutive section for an evaluation, and then, photomicrographs were obtained by a light microscope (Zeiss, Jena, Germany).

### 2.10. qRT-PCR Analysis

The total RNA was extracted with the RNAiso Plus kit (Takara, Dalian, China). RNA integration was assessed by electrophoresis on an agarose gel at a concentration of about 1%. RNA concentration was measured with NanoDrop Technologies at 260–280 nm. Reverse transcription of the total RNA was manipulated according to the PrimeScript™ RT reagent kit (Takara, Dalian, China) instruction. Then, the cDNA was kept at −20°C for further research.

Real-time fluorometric quantitative PCR was prepared using ChamQ Universal SYBR qPCR Master Mix Kit (Vazyme Biotech, Nanjing, China). Reactions were promoted in the Roche LightCycler 480 II system (Roche, Mannheim, Germany) to measure gene expression levels. The sequence of the primers of the detected gene is presented in Table 2. The previously described RT-PCR reaction system was used [35]. Reaction program was 95°C for 3 minutes; program of 95°C for 5 seconds and 60°C for 30 seconds was executed for 40 cycles. Normalize related values to 18s rRNA expression utilizing the 2^-*ΔΔ*Ct^ methodology [39].

### 2.11. Statistical Methods

All data (mean ± S.E.) were calculated with SPSS 22.0. The independent sample *t*-test was used to detect statistical differences in the means between groups of fish. One-way analysis of variance (ANOVA) was used to assess significant differences among the dietary groups. Different results were considered significant at *P* ≤ 0.05. All experimental results are displayed in means ± S.E. (standard error).

## 3. Results

### 3.1. Growth Performances and Somatic Indices

All the experimental diets were well accepted by the carp. After an eight-week feeding trial, the fish fed on high-fat or high-carbohydrate diet showed a significant decrease in FW, WGR, and SGR in Table 3. However, a decrease in growth performance parameter was remarkably attenuated (*P* < 0.05) by administration of genistein in combination with high-fat or high-carbohydrate diets (Table 3). There is no significant difference between genistein-supplemented groups and the control group in the parameters such as SGR, HSI, and VSI (*P* > 0.05). The growth performance and biological parameters were significantly affected by high-fat or high-carbohydrate diets, while the supplementation with genistein reversed the effect of HF or HC feed.

### 3.2. Composition of the Whole Fish Body and Tissue

Table 4 displays the effects of experimental diets on whole fish and muscle components. The whole-body crude lipid content in the HF group was notably elevated in comparison with that in the other groups (*P* < 0.05). However, protective administrations of genistein in the HF diet abolished the HF-elevated levels of crude lipid in whole fish. Similar results were shown in muscle; genistein supplementation in the HF diet induced an obvious decrement of the crude lipid level in muscle (*P* < 0.05). No obvious differences were found from the moisture of muscle composition among all treatments (Table 4).

### 3.3. Biochemical Parameters in the Serum and Liver

The levels of serum biochemical parameters in common carp are presented in Table 5. It reveals that the application of the HF diet in fish caused a severe increase in serum TG from 2.10 ± 0.10 in control carp to 3.67 ± 0.11 mmol/L in the HF group (*P* < 0.05). However, a decreased level of TG was observed for the genistein supplementation in the HF diet group (FG). Moreover, serum T-CHO was notably higher in the HC group (*P* < 0.05), but it was downregulated by supplement with genistein in a high-carbohydrate diet. Similar lipid parameter results were shown in the liver (Table 5). The liver TG content in the HF and HC groups was considerably increased relative to a control group, whereas the genistein supplementation group (FG and CG) was significantly decreased (*P* < 0.05). Moreover, the LDL content of the HC group was greatly reduced after genistein treatment (*P* < 0.05). However, the hepatic glycogen content in the FG and CG groups apparently increased in comparison to that of the control group.

### 3.4. Hepatic Histological Observation

**Figure 1:**
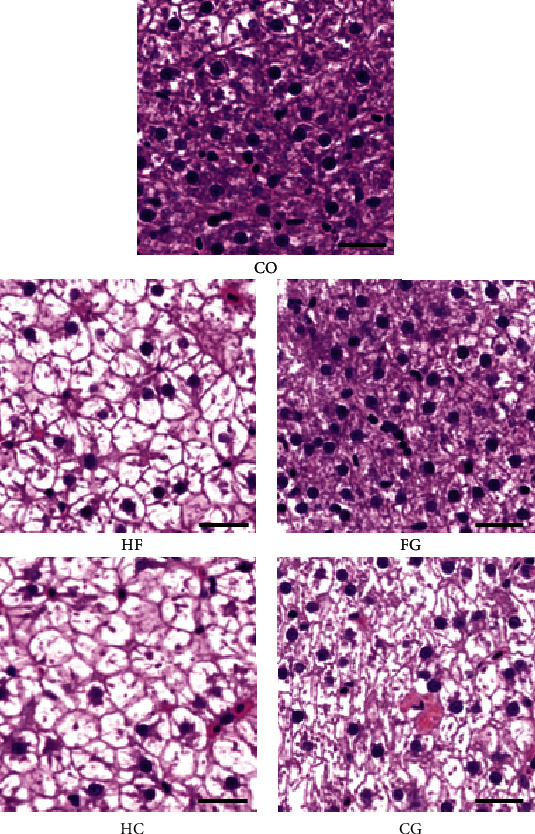
Histological characteristics of the liver of common carp fed different experimental diets (H&E stain). Bar: 20 *μ*m.

The results of liver H&E staining are shown in Figure 1. Interestingly, there is an obvious increase in lipid vacuolization and nuclear migration in the liver of carp exposed to the HF and HC diets. Moreover, the reduction in the number of nucleus occurred in the HF and HC groups. However, combined administration of 500 mg/kg genistein distinctly reduced adipocyte size of the liver compared with the HF and HC groups.

### 3.5. The Immune and Antioxidant Enzyme Activities in the Serum and Liver

**Figure 2:**
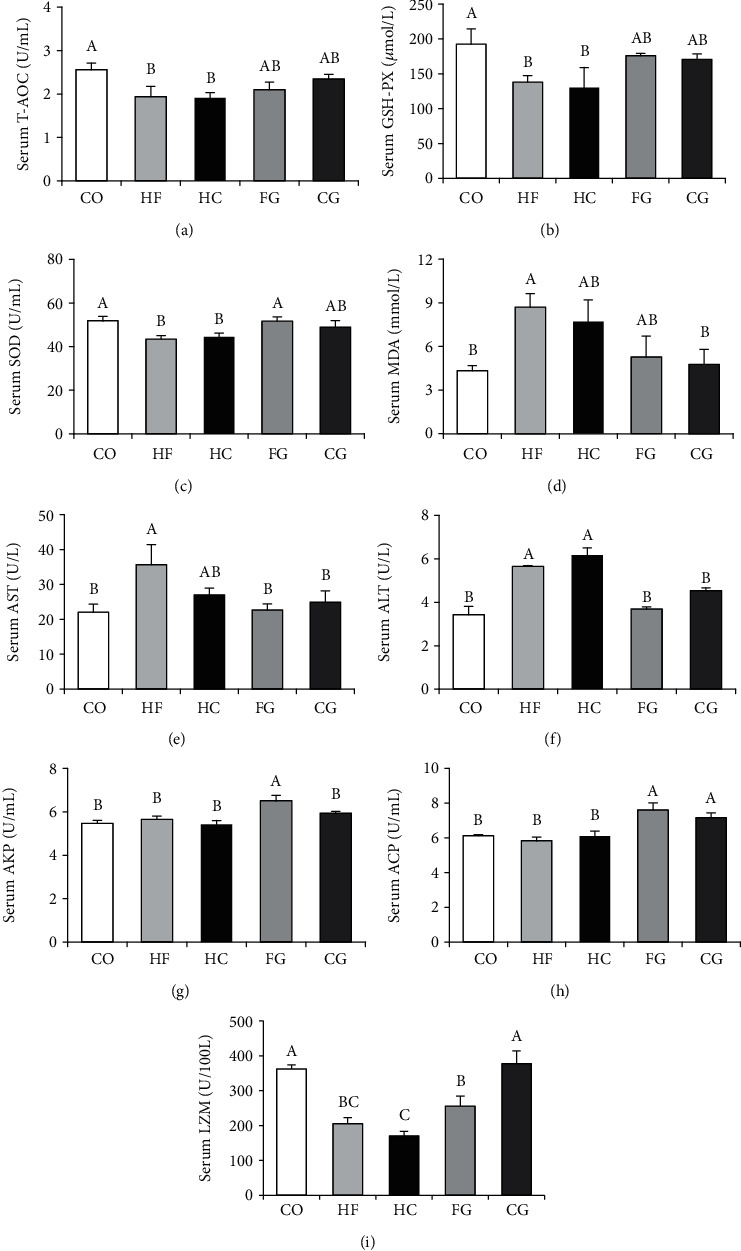
Serum antioxidant and immune enzyme activity parameters of common carp fed different experimental diets: (a) total antioxidant capacity (T-AOC); (b) glutathione peroxidase (GSH-PX); (c) superoxide dismutase (SOD); (d) malondialdehyde (MDA); (e) aspartate aminotransferase (AST); (f) alanine aminotransferase (ALT); (g) alkaline phosphatase (AKP); (h) acid phosphatase (ACP); (i) lysozyme (LZM). Values are means ± S.E. per diet in triplicate tanks (*n* = 6). The different letters indicate the differences between diets (one-way ANOVA, Duncan's test, *P* < 0.05).

The antioxidant indicators, such as enzyme activities of T-AOC, GSH-PX, and SOD, were suppressed obviously in the HF and HC groups (*P* < 0.05) in Figure 2. However, serum T-AOC, GSH-PX, and SOD returned to the same level of the control carp in administrations of genistein in HF or HC diets (FG and CG). In addition, the serum ALT and MDA contents in the HF and HC groups were higher than those in the control group, but they were significantly declined in the diet supplemented with genistein (FG and CG) (*P* < 0.05). Figure 2 reveals that serum AST activities were visibly accelerated in the HF group (*P* < 0.05); however, AST activity was decreased in the FG group which is treated with genistein (500 mg/kg) in the HF diet compared with the HF group. Furthermore, serum LZM activity was significantly declined in HF or HC groups (*P* < 0.05); however, they were upregulated in FG or CG groups. There was no significant difference of AKP and ACP among HF, HC, and control groups, but AKP and ACP were enhanced in the FG group.

**Figure 3:**
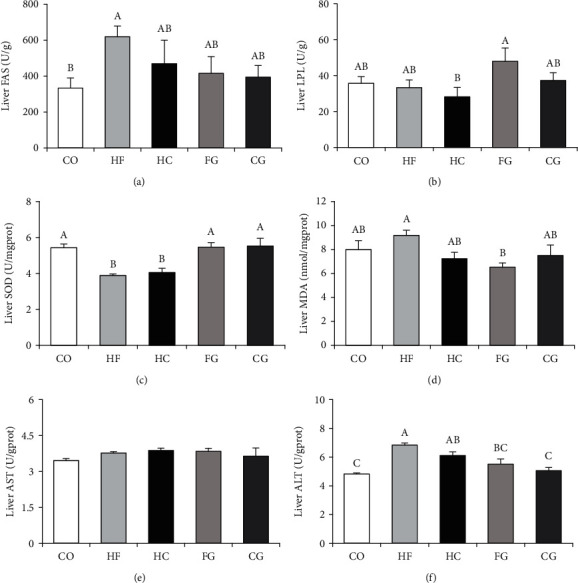
Liver enzyme activity parameters of common carp fed different experimental diets: (a) fatty acid synthetase (FAS); (b) lipoprotein lipase (LPL); (c) superoxide dismutase (SOD); (d) malondialdehyde (MDA); (e) aspartate aminotransferase (AST); (f) alanine aminotransferase (ALT). Values are means ± S.E. per diet in triplicate tanks (*n* = 6). The different letters indicate the differences between diets (one-way ANOVA, Duncan's test, *P* < 0.05).

The results of the hepatic enzyme activities are displayed in Figure 3. The activity of the FAS enzyme was significantly enhanced in HF for comparing with the CO group (*P* < 0.05). Moreover, the activities of an ALT enzyme in the HF and HC groups were also considerably higher than those in the control group. However, an increase in ALT activity was remarkably attenuated after genistein supplementation (FG and CG). On the contrary, the enzyme activity of SOD was reduced in the HF and HC groups, but it was upregulated by supplementation with genistein in high-fat or high-carbohydrate diet (FG and CG). Interestingly, the MDA content in the HF group was significantly reduced after genistein treatment (FG) (*P* < 0.05).

### 3.6. Expression of Genes Involved in Lipid Metabolism, Antioxidant Activity, and Immunity

**Figure 4:**
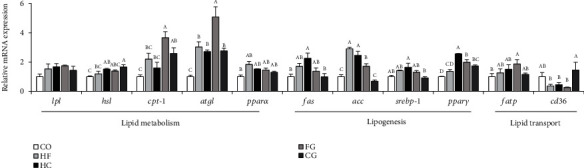
Expressions of the genes related to lipid metabolism in the liver of common carp fed different experimental diets. Values are means ± S.E. per diet in triplicate tanks (*n* = 6). The different letters indicate the differences between diets (one-way ANOVA, Duncan's test, *P* < 0.05).

The transcriptional levels of lipid metabolism genes such as *cpt-1* and *atgl* were clearly higher expressed in HF diet supplemented with genistein (FG) relative to the HF groups (Figure 4). The expression profile of genes related to lipogenesis indicated that *fas* were significantly enhanced in fish dietary with high carbohydrate (HC), whereas *fas* returned to the level of the control group after the administrations of genistein in HC diets (CG) (*P* < 0.05) (Figure 4). The decrement effect after being supplemented with genistein in high-carbohydrate diet (CG) was also shown in *acc* and *pparγ* genes (*P* < 0.05). However, *acc* and *pparγ* were significantly higher expressed in the FG group than in the HF group. In addition, the *cd36* gene level in the genistein-treated group was significantly increased compared to that in the HC group, and *fatp* expression was increased in the FG group compared to the CO group (Figure 4).

**Figure 5:**
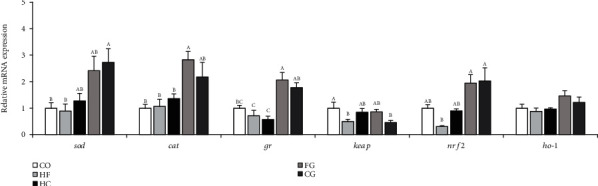
Expressions of the genes related to antioxidant in the liver of common carp fed different experimental diets. Values are means ± S.E. per diet in triplicate tanks (*n* = 6). The different letters indicate the differences between diets (one-way ANOVA, Duncan's test, *P* < 0.05).

The mRNA expressions of antioxidant-related genes are presented in Figure 5. Interestingly, the mRNA of antioxidant genes was remarkably enhanced in the fish fed with genistein-supplemented groups (FG and CG) compared with those fed with HF or HC diets. Among them, the expression of *cat* and *nrf2* genes was clearly elevated greater in the FG group than in the HF group (*P* < 0.05). The *gr* level in the FG and CG groups was considerably higher than that in the HF and HC groups (*P* < 0.05).

**Figure 6:**
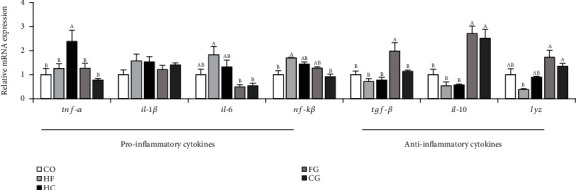
Expressions of immune-related genes in the head kidney of common carp fed different experimental diets. Values are means ± S.E. per diet in triplicate tanks (*n* = 6). The different letters indicate differences between diets (one-way ANOVA, Duncan's test, *P* < 0.05).

Changes in the expression of genes associated with immunity are presented in Figure 6. The mRNA abundance of *nf-kβ* in the HF group and *tnf-α* in the HC group is significantly upregulated when compared to that in the control group, which belonged to the proinflammatory cytokine-related genes. However, a significant downregulation of *tnf-α* and *il-6* was reported in the genistein treatment group (*P* < 0.05). In regard to anti-inflammatory cytokine-related genes, the levels of *tgf-β* and *lyz* in the FG group treated with genistein were remarkably enhanced compared with those in the HF group (*P* < 0.05). In addition, the transcriptional level of *il-10* was remarkably promoted in the genistein addition group (FG and CG) relative to the HF and HC groups (*P* < 0.05).

## 4. Discussion

In the present experiment, the growth parameter WGR was significantly reduced in the HF and HC groups by comparison with the basic diet group (CO). This is consistent with several studies on long-term diets, where high-fat or high-carbohydrate diets resulted in the reduction of growth performance in various species of fish [10, 17, 40–43]. Noticeably, the supplementation of genistein in the HF and HC groups increased the final weight, WGR, and SGR, indicating that genistein could alleviate the adverse impacts that an HC or HF diet can have on common carp. However, we found no significant overall differences compared to the control group after treatment with genistein. A similar effect of genistein on growth performance has been reported with a normal diet in previous research by our lab [35] and by Pastores team in research on rainbow trout [44]. The same phenomenon has also been reported in mice [28]. Previous findings in conjunction with our current research show that the promotive effect of genistein on growth performance may be affected by the fish nutrition background.

Various studies have reported that long-term intake of high-carbohydrate and high-lipid diets can lead to hepatic lipid deposition in different fish species [15, 40, 45, 46]. Consistent with these results, this study also found that HF and HC diets resulted in significant lipid accumulation in common carp, as evidenced by severe lipid vacuolization in the hepatocytes and both high crude lipid and high levels of HSI and VSI in whole fish, as well as high TG in serum and the liver after the eight-week feeding trial. In addition, HSI and VSI are commonly used to assess the nutritional status of fish. It is generally assumed that the intake of a high nutrient diet leads to an increasing trend, which is used to predict impaired liver function and an association with poor health and growth restriction [47]. Based on the liver H&E staining results, the fish in the HF and HC groups exhibited an increase in the number of cell vacuoles and the amount of nucleus translocation, as well as a decrease in the number of nuclei. Similar reports were observed in largemouth bass (*Micropterus salmoides*) [42], Nile tilapia [10], and grass carp [48] that were fed with high-fat or high-carbohydrate diets. Moreover, serum indicators are useful indexes for assessing the nutritional status of fish, reflecting the physiological and metabolic function. The serum results showed that the contents of T-CHO, HDL-C, and LDL-C decreased in the HF group, compared to the control group. In addition, the TG, T-CHO, and HDL-C in serum were also elevated in the HC group, which is the characteristic of hyperlipidemia. Previous reports suggest that both excess dietary carbohydrates and fats lead to lipid deposits in carp [49]. All these findings suggest that continuous feeding of high-carbohydrate and high-fat diets can lead to hyperlipidemia and hypercholesterolemia in common carp. Similarly, this abnormal amount of lipid has also been observed in large yellow croaker (*Larimichthys crocea*) [14, 50], silvery-black porgy juveniles (*Sparidentex hasta*) [51], and hybrid grouper (*Epinephelus lanceolatus*♂ × *E. fuscoguttatus*♀) [52].

Genistein, the primary form of soy isoflavone, has received a great deal of attention as a nutritional additive for the treatment of metabolic problems [53]. In the current investigation, it was notable that genistein supplementation greatly decreased lipid levels in whole fish, serum, and liver tissue. A similar result was observed in nonalcoholic fatty liver rats, in which genistein reduced abnormal lipid metabolism, liver damage, and liver weight [54]. The main target of genistein may be the liver. Therefore, to learn more about genistein's function on lipid metabolism, we examined enzyme activities of lipid metabolism and mRNA abundance of genes relevant to the hepatic lipid synthesis, transport, and lipolysis. The FAS activity was notably enhanced in the HF and HC groups while the LPL had the opposite trend when compared to the control group. Moreover, the dietary treatment with genistein upregulated the mRNA expressions of several lipolysis genes such as *hsl*, *cpt-1*, and *atgl*, but the lipid synthesis genes (*fas*, *acc*, and *srebp-1*) were downregulated. It is indicated that genistein at the concentration of 500 mg/kg supplementation in diet enhanced lipolysis in common carp. Genistein had the same effect on modulating hepatic lipid metabolism in rainbow trout [33, 55]. Similarly, genistein has also been found to reduce hyperlipidemia and ameliorate a fatty liver, by reducing lipid deposition, improving lipid metabolism, and reducing liver steatosis in studies on mice [53, 56, 57]. The expression level of *cd36*, a gene related to fatty acid transport, was enhanced in the genistein group but not in the HC group. Furthermore, the enhancement effect of genistein on fatty acid *β*-oxidation, lipid transport, and cholesterol metabolism in the liver has also been reported in chickens [31, 58]. In the current study, these findings indicated that the lipid-lowering effect of dietary genistein on carp fed with HF and HC diets may be caused by an increase in lipid breakdown genes as well as a decrease in lipid synthesis genes. Genistein is an effective lipid-lowering additive in common carp that can be used to combat the negative consequences of high-carbohydrate and high-fat diet loads.

Blood biochemical indicators can reflect the physiological response of fish to nutritional status. Both ALT and AST are used as common indicators to assess the health of fish livers. Their increased activity indicates liver disease and impaired function because these enzymes can get into the bloodstream through damaged liver cell membranes [59, 60]. In our study, the serum ALT and AST activities were clearly elevated in the HF and HC groups, but dietary genistein reduced their levels. Similarly, dietary genistein supplementation reduced serum AST and ALT activities in mice fed with HF or HC diets [61–63]. Moreover, genistein can also act as an enhanced antioxidant and has been studied extensively. T-AOC, GSH-PX, and SOD are classic antioxidant enzymes that can preserve cells and tissues from peroxidation [64]. In this study, T-AOC, GSH-PX, and SOD clearly declined in the HF and HC diets, while their activities were enhanced after genistein treatment, indicating its beneficial effects on health status. Furthermore, the dietary supplementation of genistein upregulated the mRNA abundance of several antioxidant-related genes (such as *sod*, *cat*, *gr*, and *nrf2*). Additionally, MDA content also increased relative to the control group. The MDA index is the decomposition product of lipid peroxidation caused by free radicals in the body. The level of its content reflects the health status of fish. The higher the content, the more serious the damage to the body. Our study also demonstrated that high-carbohydrate and high-fat diets cause oxidative damage to the liver, similar to previous findings on fish [12, 16, 48, 65–67]. Genistein supplementation can improve antioxidant status and enhance the oxidant defense capability [68]. This is consistent with the results in golden pompano (*Trachinotus ovatus*) [19], juvenile grass carp [69], and mammals such as young piglets [70].

Several studies report that oxidative stress caused by increased lipid accumulation in fish that have been fed with a high-energy diet can induce an inflammatory response [71–75]. Serum AKP, ACP, and LZM activities are considered to be the key components of the nonspecific immune system in fish. In the present study, high dietary carbohydrate and fat levels significantly decreased the activity of LZM, similar to observations in previous studies [21, 76, 77]. However, the activities of AKP, ACP, and LZM in serum were notably increased by genistein supplementation in common carp, indicating its beneficial effects on health status. Moreover, the head kidney is an important immune organ in fish. Our present experiment indicated that the transcription level of proinflammatory cytokines (*tnf-α*, *il-6*, and *nf-kβ*) was downregulated after genistein treatment. On the contrary, the transcriptional level of anti-inflammatory cytokines, *tgf-β* and *il-10*, was upregulated in the head kidney by genistein supplementation. Similar to our results, inflammatory responses were induced by an HF diet in C57BL/6 mice, which could be attenuated by combining dietary genistein and exercise [78]. In addition, supplementation with soy isoflavones has been shown to activate adaptive immune system pathways and improve the immune status in pigs [79–81]. Similarly, the expression of immune-related genes and resistance to pathogenic bacteria (*Aeromonas hydrophila* or *Vibrio harveyi*) could be enhanced after supplementation of soy isoflavone in grass carp [69], common carp [35], and juvenile golden pompano [19]. Therefore, genistein treatment can enhance the immune response to high-carbohydrate and high-fat dietary loads in common carp.

## 5. Conclusions

**Figure 7:**
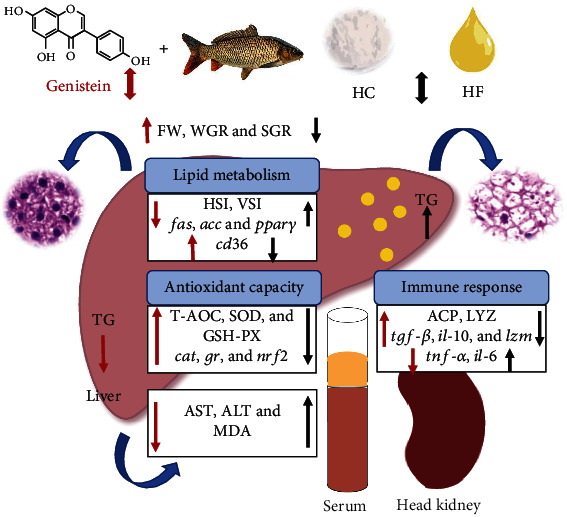
Summary graph.

In summary, hepatic lipid and oxidative stress increased in common carp under a high-carbohydrate and high-fat dietary load. In addition, the activity of inflammatory factors in the head kidney was enhanced while the mRNA abundance of proinflammatory factor genes increased and anti-inflammatory factor genes decreased. However, dietary supplementation of 500 mg/kg genistein can effectively alleviate hepatopancreas injury and lipid accumulation in carp fed with HF and HC diets by enhancing lipolysis, improving the antioxidant capacity, and supporting the immune system (Figure 7).

## Figures and Tables

**Table 1:** Ingredient and proximate analysis of experimental diets (g/kg).

Ingredients (%)	CO	HF	HC	FG	CG
Fish meal	16	16	16	16	16
Meat and bone meal	4	4	4	4	4
Corn gluten feed	28	28	28	28	28
Wheat flour	15	15	15	15	15
Corn starch	14.4	14.4	29.7	14.4	29.7
Cellulose microcrystalline	15.6	9.9	0.3	9.9	0.3
Choline chloride	0.5	0.5	0.5	0.5	0.5
CMC	1.5	1.5	1.5	1.5	1.5
Corn oil	1.095	6.795	1.095	6.795	1.095
Linseed oil	0.905	0.905	0.905	0.905	0.905
Vitamin premix	1	1	1	1	1
Mineral premix	2	2	2	2	2
Genistein	0	0	0	0.05	0.05
Proximate composition (% dry matter)					
Dry matter	93.89	93.32	92.50	93.41	93.59
Protein	32.59	32.59	32.63	32.59	32.63
Lipid	5.44	11.03	5.47	11.03	5.47
Ash	10.05	10.42	10.44	10.30	10.53

^1^Vitamin mixture (g kg^−1^ of premix): vitamin A: 800000 I.U.; vitamin D3: 160000 I.U.; vitamin E: 15 g; vitamin K: 3.325 g; vitamin B1: 1.5 g; vitamin B2: 1.25 g; vitamin B6: 1.1 g; vitamin B12: 0.004 g; vitamin C: 2.5 g; creatine: 5.5 g; folic acid: 0.070 g; biotin: 0.125 g; nicotinic acid: 4 g; D-pantothenic acid: 4.5 g. ^2^Mineral mixture (mg kg^−1^ of premix): P: 105000 mg; Ca: 330000 mg; Mg: 45000 mg; Fe: 15000 mg; I: 50 mg; Se: 9 mg; Cu: 350 mg; Zn: 3000 mg; Mn: 1500 mg; Co: 11 mg.

**Table 2:** The primers were used in quantitative real-time PCR in this experiment.

Gene	Forward and reverse primer (5′-3′)	Amplification efficiency (percent)	GenBank accession no.
*lpl* F	CGCTCCATTCACCTGTTCAT	91.4	FJ716101.1
*lpl* R	GCTGAGACACATGCCCTTATT		
*hsl* F	ATGATTTGGATGCGCAGACC	103.6	MF061228.2
*hsl* R	AAACGCTCCAGTGCAGTTTG		
*cpt-1b* F	CAGATGGAAAGTGTTGCTAATGAC	92.0	JQ361077.1
*cpt-1b* R	TGTGTAGAAGTTGCTGTTGACCA		
*atgl F*	CACCAACACCTCCATTCAGTTCACA	92.3	KY906167.1
*atgl* R	ACTCTTCATCCTCCTCACCGTCAG		
*pparα* F	GCGTGCTTTGGCTTTGTT	91.2	FJ849065.1
*pparα* R	GGGAAAGAGCAGCACGAG		
*fas* F	GACAGGCCGCTATTGCTATT	95.5	GQ466045.1
*fas* R	TGCCGTAAGCTGAGGAAATC		
*acc-1* F	GTCACTGGCGTATGAGGATATT	95.8	XM_042757417.1
*acc-1* R	TCCACCTGTATGGTTCTTTGG		
*srebp-1c* F	CGTCTGCTTCACTTCACTACTC	97.8	XM_042730308.1
*srebp-1c* R	GGACCAGTCTTCATCCACAAA		
*pparγ* F	AAGTCACCGAATTCGCCAAG	99.52	FJ849064.1
*pparγ* R	TGCCGTCTTTGTTCATGAGG		
*fatp* F	AATCAGCCGAAGAAGGACAC	103.5	XM_019081506.1
*fatp* R	AATCCACAGGCACCCGTCTT		
*cd36* F	GGAGAACCCAGATAACC	96.7	KM030422.1
*cd36* R	CTGCCATAGAGGAAGTG		
*tnf-α* F	GTGTCTACAGAAACCCTGGA	93.0	XM_019088899.2
*tnf-α* R	AGTAAATGCCGTCAGTAGGA		
*Il-1β* F	TTACAGTAAGACCAGCCTGA	94.4	XM_019080073.2
*Il-1β* R	AGGCTCGTCACTTAGTTTGT		
*Il-6* F	GCAGCGCATCTTGAGTGTTTAC	96.8	XM_019110666.2
*Il-6* R	CTGCTGCTCCATCACTGTCTTC		
*nf-kβ* F	AATGTGGTGCGTCTGTGCTT	97.2	XM_019094112.1
*nf-kβ* R	TGTTGTCATAGATGGGGTTGGA		
*tgf-β* F	ACGCTTTATTCCCAACCAAA	103.6	AF136947.1
*tgf-β* R	GAAATCCTTGCTCTGCCTCA		
*Il-10* F	CTCCGTTCTGCATACAGAGAAA	91.8	XM_019092454.1
*Il-10* R	TCATGACGTGACAGCCATAAG		
*lyz* F	GTGTCTGATGTGGCTGTGCT	99.8	XM_019104788.2
*lyz* R	TTCCCCAGGTATCCCATGAT		
*sod* F	CGCACTTCAACCCTCAT	96.4	XM_019111527.2
*sod* R	CATTGCCTCCTTTACCC		
*cat* F	TTCCTGTGGGACGCCTTGT	95.1	JF411604.1
*cat* R	TCCGAGCCGATGCCTATGT		
*gr* F	TGGCTGGTATCCTTTCC	104.1	XM_019102099.1
*gr* R	TGTCGTCAGGGTCTTTT		
*keap* F	CAGTGGGCGAGAAGTGT	91.4	JX470752.1
*keap* R	TTTGATGGCTCCAGGTT		
*nrf2* F	ACGACAAATGCCGAAGT	93.4	JX462955.1
*nrf2* R	CTGCCTCATCTAGTGGAAA		
*18S* F	GAGACTCCGGCTTGCTAAAT	94.1	FJ710826.1
*18S* R	CAGACCTGTTATTGCTCCATCT		

**Table 3:** Growth performance and biological parameters of common carp fed different experimental diets.

Index	CO	HF	HC	FG	CG
IW (g)	6.36 ± 0.12	6.37 ± 0.20	6.39 ± 0.21	6.35 ± 0.22	6.35 ± 0.16
FW (g)	15.33 ± 0.43^a^	13.04 ± 0.38^c^	13.91 ± 0.44^bc^	13.27 ± 0.39^abc^	14.63 ± 0.41^ab^
WGR (%)	135.86 ± 1.26^a^	104.65 ± 0.32^d^	117.63 ± 2.21^c^	119.40 ± 0.58^c^	130.54 ± 1.49^b^
SGR (% day^−1^)	1.51 ± 0.18^a^	1.26 ± 0.05^c^	1.35 ± 0.05^bc^	1.42 ± 0.29^ab^	1.45 ± 0.05^ab^
HSI (%)	2.15 ± 0.16^c^	2.90 ± 0.14^a^	2.81 ± 0.15^ab^	2.32 ± 0.12^bc^	2.11 ± 0.22^c^
VSI (%)	6.66 ± 0.21^b^	8.88 ± 0.58^a^	8.71 ± 0.29^a^	7.15 ± 0.21^b^	6.35 ± 0.32^b^
SR (%)	96.67 ± 1.36	96.67 ± 2.7	98.33 ± 1.36	100.00	100.00

IW and FW: initial and final body weight; WGR: weight gain rate; SGR: specific growth rate; HSI: hepatosomatic index; VSI: viscerosomatic index; SR: survival rate. Values are means ± S.E. per diet in triplicate tanks (*n* = 9). The different letters indicate differences between diets (one-way ANOVA, Duncan's test, *P* < 0.05).

**Table 4:** The body composition of common carp fed different experimental diets.

Index	CO	HF	HC	FG	CG
Whole fish (%)					
Moisture	73.90 ± 0.18^b^	73.06 ± 0.58^c^	76.17 ± 0.04^a^	72.57 ± 0.09^c^	71.30 ± 0.08^d^
Crude lipid	32.49 ± 0.87^bc^	38.56 ± 0.17^a^	33.47 ± 1.00^bc^	35.30 ± 0.19^b^	31.31 ± 0.79^c^
Crude protein	51.86 ± 0.93	49.79 ± 0.75	53.33 ± 2.29	49.21 ± 2.17	49.27 ± 0.58
Ash	12.20 ± 0.83^b^	13.16 ± 0.49^b^	15.55 ± 0.18^a^	12.31 ± 0.05^b^	10.86 ± 0.39^c^
Muscle (%)					
Moisture	79.93 ± 0.10	79.83 ± 0.11	79.65 ± 0.54	79.84 ± 0.66	79.77 ± 0.52
Crude lipid	11.06 ± 0.30^ab^	12.85 ± 0.92^a^	12.36 ± 0.55^ab^	10.23 ± 0.94^b^	12.19 ± 0.76^ab^
Crude protein	82.74 ± 1.50^a^	79.69 ± 0.82^ab^	76.64 ± 1.24^b^	80.06 ± 2.17^ab^	77.01 ± 1.92^ab^
Ash	6.32 ± 0.09^b^	6.25 ± 0.08^b^	6.10 ± 0.11^b^	6.31 ± 0.11^b^	7.27 ± 0.41^a^

Values are means ± S.E. per diet in triplicate tanks (*n* = 6). The different letters indicate the differences between diets (one-way ANOVA, Duncan's test, *P* < 0.05).

**Table 5:** Serum and liver biochemical indicators of common carp fed different experimental diets.

Index	CO	HF	HC	FG	CG
Serum					
Glucose (GLU, mmol/L)	3.26 ± 0.42^ab^	3.95 ± 0.26^a^	3.04 ± 0.33^ab^	2.79 ± 0.23^b^	3.29 ± 0.06^ab^
Total cholesterol (T-CHO, mmol/L)	4.51 ± 0.24^b^	3.71 ± 0.18^c^	6.11 ± 0.19^a^	4.45 ± 0.21^bc^	5.07 ± 0.41^b^
Triglycerides (TG, mmol/L)	2.10 ± 0.10^c^	3.67 ± 0.11^a^	3.57 ± 0.18^ab^	3.04 ± 0.22^b^	3.24 ± 0.10^ab^
Low-density lipoprotein cholesterol (LDL-C, mmol/L)	1.24 ± 0.18^ab^	1.08 ± 0.96^b^	1.25 ± 0.19^ab^	1.65 ± 0.04^a^	1.24 ± 0.23^ab^
High-density lipoprotein cholesterol (HDL-C, mmol/L)	1.93 ± 0.06^ab^	1.49 ± 0.31^b^	2.07 ± 0.30^ab^	2.32 ± 0.22^ab^	2.49 ± 0.12^a^
Liver					
Total cholesterol (T-CHO, mmol/gprot)	0.0646 ± 0.0025	0.0634 ± 0.0011	0.0671 ± 0.0006	0.0641 ± 0.0009	0.0694 ± 0.0028
Triglycerides (TG, mmol/gprot)	0.0554 ± 0.009^c^	0.1649 ± 0.004^a^	0.1118 ± 0.0154^b^	0.0650 ± 0.0083^c^	0.0535 ± 0.0073^c^
Low-density lipoprotein cholesterol (LDL-C, mmol/gprot)	0.0209 ± 0.0015^c^	0.0355 ± 0.0080^bc^	0.0573 ± 0.0045^a^	0.0252 ± 0.0044^bc^	0.0399 ± 0.0051^b^
High-density lipoprotein cholesterol (HDL-C, mmol/gprot)	0.0062 ± 0.0014^ab^	0.0038 ± 0.0012^b^	0.0057 ± 0.0006^ab^	0.0091 ± 0.0029^ab^	0.0110 ± 0.0019^a^
Hepatic glycogen (mg/g)	25.37 ± 1.20^b^	28.15 ± 1.00^ab^	27.11 ± 1.32^ab^	29.04 ± 0.75^a^	28.95 ± 0.40^a^

Values are means ± S.E. per diet in triplicate tanks (*n* = 6). The different letters indicate the differences between diets (one-way ANOVA, Duncan's test, *P* < 0.05).

## Data Availability

On a reasonable suggestion, the corresponding author will provide the information supporting the study's conclusions.
